# Social harassment induces anxiety-like behaviour in crayfish

**DOI:** 10.1038/srep39935

**Published:** 2017-01-03

**Authors:** Julien Bacqué-Cazenave, Daniel Cattaert, Jean-Paul Delbecque, Pascal Fossat

**Affiliations:** 1 INCIA - Institut de Neurosciences Cognitives et Intégratives d’Aquitaine, University of Bordeaux, CNRS UMR 5287, 146 Rue Leo Saignat, 33076 Bordeaux, France

## Abstract

Social interactions leading to dominance hierarchies often elicit psychological disorders in mammals including harassment and anxiety. Here, we demonstrate that this sequence also occurs in an invertebrate, the crayfish Procambarus clarkii. When placed in the restricted space of an aquarium, crayfish dyads generally fight until one of the opponents suddenly escapes, thereafter clearly expressing a submissive behaviour. Nevertheless, the winner frequently keeps on displaying excessive aggressive acts, having deleterious consequences in losers and interpreted as harassment behaviour. We indeed observed that, contrary to winners, losers expressed anxiety-like behaviour (ALB) in correlation with the stress intensity they suffered during the harassment period mainly. Injections of an anxiolytic abolished ALB, confirming its homology with anxiety. A serotonin (5-HT) antagonist had the same effect, suggesting a role for 5-HT, whose brain concentrations increased much more in losers than in winners. Our findings suggest that the bases of harassment and of its anxiogenic consequences have emerged very early during evolution, and emphasize crayfish as an unexpected but potentially fruitful model for the study of these social disorders.

Agonistic interactions, including aggression and fights, are prerequisites for the development of social hierarchies, allowing animals to delimit their territories and/or to share common resources. However, these interactions may represent sources of deep social stress that can alter physiology and cause severe psychiatric disorders^1^,^2^. Exaggerated aggressive acts, such as violence or harassment, may lead to generalized anxiety or depression in victims. Although this behavioural sequence strongly impacts human life, animal models are necessary for use in experimental studies to investigate the fundamental bases of behaviour^1^,^2^,^3^. Such models, typically involving primates or rodents, have been chosen because they are closely related to humans. Invertebrates, albeit outstanding models for the study of aggression^4^,^5^, have not yet been considered for the study of aggression-related disorders. However, they share many ancestral neurobiological mechanisms with humans, including those related to emotions^6^,^7^,^8^,^9^ or pain^10^. In particular, anxiety-like behaviour (ALB) was recently observed in crayfish after physical stress^7^. As clawed crustaceans are well-studied models of agonistic behaviour^4^,^11^,^12^,^13^, we aimed to investigate whether and how social interactions could also elicit ALB in crayfish. For this purpose, we examined ALB after social pairing and found it related not only to defeat but also to repetitive hostile behaviour displayed by the winner after its victory. This behaviour, similar to that frequently observed in humans and primates^14^,^15^, was interpreted as psychological harassment.

Crayfish can live either alone in territorial conditions, when space is available, or together in groups, when animals are compelled to gather in limited areas (e.g., if water runs out). Isolated animals very aggressively defend their territory; in contrast, aggression diminishes when animals are grouped^12^. Experimentally, when pairs of size-matched *P. clarkii* males that had been previously isolated for at least 2–3 weeks are placed together in a confined area, they generally display the following typical behavioural sequence: after a brief aquarium exploration, the animals come face to face, and their aggression rapidly escalates into a series of fights (Fig. 1). Even if the animals have similar sizes and strengths, fights end with the decision of one of the protagonists to escape the fight by retreating, frequently by swimming backward with a sudden tail flip. Notably, while the loser instantaneously stops behaving aggressively toward its protagonist, the winner continues to attack for an extended time, probably because its adversary remains within reach in the aquarium. The winner’s behaviour (see Fig. 1 and videos in the supplementary section), which has not been previously considered in invertebrates, is a repetitive hostile behaviour resembling psychological harassment in humans.

We estimated the ALB level of the winning and losing male crayfish after a 20-min pairing by measuring their light avoidance behaviour in a dark/light plus maze^7^,^16^. The results in Fig. 2 show that ALB significantly increased in the losers but not in the winners (Fig. 2B–C). The main variables that were used to characterize ALB in crayfish^7^, namely the percentage of time spent in the maze’s illuminated arms and the retreat ratios at the dark/light limits, were significantly different between the winners and losers at the end of experiment. These differences were also confirmed by a principal component analysis (PCA) that was performed on a total of 7 unrelated variables measured in the dark/light plus maze (Fig. 2C3 and Sup Figs 1–2).

The avoidance behaviour after defeat was suppressed (Fig. 2D) by injecting losers with chlordiazepoxide (CDZ). Similarly with our observations after physical stress, taking into account that this specific anxiolytic drug has no effect on isolated or unstressed crayfish^7^, the suppression of social ALB in losers confirms that it is homologous to human anxiety. Also consistent with our previous observations with physical stress^16^, social ALB was clearly correlated with the intensity of stress that was suffered by the losers, i.e., stress caused by the aggressive acts of the winners. Figure 3 shows that the ALB intensity in the losers was correlated with the approaches and attacks of the winners throughout the entire pairing period (Fig. 3A and Sup Fig. 3A). Surprisingly, however, this correlation was essentially caused by the attacks and approaches that occurred after defeat, i.e., during the harassment period (Fig. 3B and Sup Fig. 3B), and not by the fight duration or by the attacks occurring during the fighting period (Fig. 3C,D and Sup Fig. 3C,D). These observations demonstrate the effect of the winners’ harassment behaviour and also suggest that social ALB is mainly controlled downstream of the decision to flee.

Again, in agreement with our previous observations after physical stress^7^, the loser’s ALB was associated with an increased 5-hydroxytryptamine (5-HT) concentration in the brain (Fig. 4). This concentration, measured using high-performance liquid chromatography (HPLC) after a 20-min pairing, was significantly higher in the losers than in isolated animals (i.e., before-pairing controls). We also observed a rise of 5-HT concentrations in winners, which however remained significantly lower than in losers (Fig. 4A). The higher level of 5-HT in losers led us to consider its involvement in social ALB, as previously observed after physical stress^7^. This hypothesis was confirmed by injecting a 5-HT antagonist, before pairing, into the smaller animal of each pair, which is generally considered the most probable loser^11^. Methysergide, a broad-spectrum 5-HT antagonist, prevented ALB in defeated animals: the time that these animals spent in the light arms of the maze and their retreat ratios were significantly different from those of the non-injected losers (Fig. 4B,C). PCA confirmed that methysergide-injected crayfish were different from the saline controls (Fig. 4D and Sup Fig. 2). These experiments thus confirmed that ALB is not a prerequisite for the loser to stop fighting and to retreat. They also demonstrated that 5-HT and 5-HT receptors are involved in the control of social ALB during agonistic interactions in crayfish, confirming our previous observations after physical stress^7^. Recent studies in mice have also emphasized the role of 5-HT in the control of anxiety by revealing specific anxiogenic circuits^17^. However, such circuits remain to be described in crayfish.

Altogether, our results demonstrate that social interactions in crayfish can induce ALB in defeated animals. Similar to our previous observations after physical stress^7^, ALB after social stress involves an increase in the brain concentration of serotonin. Interestingly, 5-HT not only can induce ALB^7^ but also can control aggression in crayfish by affecting the decision to retreat^11^. However, our present results show that methysergide inhibited ALB in animals that nevertheless retreated. Thus, ALB appears to be rather a consequence of than a prerequisite for defeat. It can be inferred that the serotonin pathways controlling ALB and aggression are distinct and relatively autonomous, possibly sensitive to different 5-HT concentrations, as suggested the higher concentrations observed in the brain of losers compared to winners.

Moreover, our observations show that the intensity of ALB is not related to the intensity of the adversary attacks occurring during the fighting period but mainly results from post-fighting period harassment. Our results thus suggest that crayfish have a sense of defeat/victory and that they display either social ALB or harassment behaviour depending on the outcome of the fights. These two behaviours are probably exacerbated under our experimental conditions, which forced these territorial animals to remain in a confined space. Under natural conditions, these animals would undoubtedly immediately distance themselves from one another. The question remains as to whether the harassment behaviour described here in crayfish is homologous to psychological harassment in humans and in some primates^14^,^15^. Both types of harassment have similar characteristics (hostile, repetitive and deleteriously affecting the target animal), and the former can be interpreted as a consequence of the winner’s failure to chase its opponent out of the aquarium, i.e., the unsatisfactory result of its domination.

In conclusion, despite rudimentary social interactions, crayfish have a sense of defeat/victory and can display either an emotion homologous to anxiety or an exaggerated aggression, the latter of which has similar features as human psychological harassment. ALB and aggression are both modulated by 5-HT in these crustaceans, and these behaviours are sensitive to pharmaceutical drugs that are administered to humans with psychiatric disorders. Our observations potentially suggest crayfish as unexpected models for studying such behaviours and related disorders, including psychological harassment.

## Methods

### Animals

We used male crayfish (*Procambarus clarkii*), averaging 8.7 ± 0.2 cm in length and 22 ± 1 g in weight. Crayfish were fished in swamps near Bordeaux, at the “Réserve naturelle de Bruges”, and stored in individual tanks (50 × 30 × 30 cm) equipped with circulating water, inside a specific animal house at 20 °C with a 12 h/12 h light/dark cycle. They were fed pellets *ad libitum*. Each experimental animal was isolated during at least 3 weeks before any experiment in order to erase past life histories, including previous social interactions. All experiments were performed in accordance with the CNRS guidelines for animal care.

### Social interactions

Social interactions were performed in a specific aquarium (L × l × h = 33 × 23 × 20 cm, see Videos). We paired males with a 5–20% weight difference. They were placed together in the fight aquarium for 20 minutes. Agonistic behaviors were recorded with a video camera (Canon HDV) placed laterally, the aquarium being illuminated from above. During the experiment, we particularly measured two aggressive behaviors, the number of approaches and the number of attacks, and two defensive behaviors, the number of escapes and the number of tail flips. An approach is recognized when one crayfish is moving toward its congener, but without contact. An attack is accounted when the animal ends an approach by accelerating with elevated claws until touching or grasping its opponent. An escape is accounted when a protagonist avoids its approaching or attacking opponent by forward or backward locomotion. A tail flip is a rapid escape by backward swimming.

### ALB analysis: the dark/light plus maze protocol

The objective of this protocol, described in details elsewhere^7^,^18^, was to analyze the spontaneous exploration behavior of crayfish confronted with a novel environment. The aquatic dark/light plus maze (total dimensions: 60 × 60 cm) comprises two dark arms (light intensity, 10 lux) and two illuminated arms (light intensity, 50 lux). Each arm was 25 cm in length and 10 cm in width. Animals were tested individually and only once in the maze. Each tested crayfish was first placed in the center of the arena and confined for one minute under a small opaque chamber. After this delay, the animal was released, and its exploratory behavior was recorded with a video camera (Sony Inc.) placed above the arena. We used Ethovision software XT8 (Noldus, NL) to detect and track crayfish in the arena.

### Serotonin measurements

5-HT was measured using reverse phase high performance liquid chromatography with electrochemical detection (RP-HPLC-ECD) as previously described^7^. Crayfish brains were rapidly extirpated and weighed, then separately homogenized in 200 μL of 0.1 N HClO_4_ by sonication and centrifuged at 13000 rpm for 30 min at 4 °C. Aliquots of 10 μl of the supernatants were injected into RP-HPLC column (Chromasyl Stability C8, 150 × 4.6 mm). The mobile phase (70 mM NaH_2_PO_4_, 0.1 mM disodium EDTA, 2 mM sodium octane-1-sulfonate monohydrate, 7% methanol, pH 3.9) was delivered at 1 mL/min by a Beckman 128 pump. Serotonin detection was performed using a coulometric detector (Coulochem II, ESA) equipped with a dual-electrode analytical cell (5011 analytical cell, ESA; potentials set at +350 and −270 mV respectively). Known quantities (5–1000 pg) of reference compounds allowed detector calibration and bioamine measurements in brain extracts. Linearity of the detector was verified from 5 to 1000 pg. Sensibility of detection in biological samples was equal or inferior to 10 pg, but aliquots containing at least 50 pg were generally injected. Each individual extract was analyzed at least in duplicate to check for the reproducibility of results. Due to the very simple extraction procedure and to the convenient stability (over months) of our coulometric detector, the use of an internal standard, which was found to interfere with endogenous unknown compounds, was not retained in our protocol. Results were expressed as pg of 5-HT per mg brain fresh weight.

### Drug treatments

Methysergide maleate salt (5-HT1 and 5-HT2 blocker) and chlordiazepoxide hydrochloride (CDZ), purchased from Sigma Aldrich (St Louis, MO, USA), were dissolved in crayfish saline (195 mM NaCl, 5 mM KCl, 13 mM CaCl_2_, 2 mM MgCl_2_ and 3 mM HEPES, pH 7.65). Crayfish were injected intramuscularly, between two abdominal segments. Methysergide was used at 5 μg per g fresh weight and injected 10 min before agonistic interactions, whereas CDZ was injected at 15 μg/g immediately after the 20-min interactions.

### Statistical analyses

Comparisons of means ± SEM were obtained and plotted using Prism software (GraphPad, US). Non-parametric Mann-Whitney tests were used to compare 2 groups, and Kruskal-Wallis tests, followed by Dunn’s multiple comparison tests, were used for more than 2 groups. A value of *P* < *0.05* was considered significant (*P < 0.05, **P < *0.01* and ***P < *0.001).*

Steiger’s Z-test was used to compare two dependent correlation coefficients with one common variable.

Principal component analysis was performed as previously described^7^ using R software (Ade4 package) based on the behavioral variables described in Sup Table 1. In a first step, the contribution of each variable to the variance in the first and second components of the PCA was calculated (Sup Fig. 1A). We only used the variables whose contribution to the variance of a given component was larger than the mean contribution of all variables to that component. Percentage of time in light arms and retreat ratios were mostly responsible for variance in the first component (Sup Fig. 1A). This analysis indicates that the first component of the PCA (horizontal axis) mostly represented avoidance of light arms. On the left side are animals that spent substantial time in the light arms; on the right are animals that hesitated to enter the light arms (large latency and high level of retreat, a behavior characteristic of anxious animals). Therefore, the first component of the PCA seems mostly indicative of the anxiety level.

In the second step, the location of each animal group was represented on the plane defined by the PCA (Sup Fig. 1B). The separation between pairs of groups was evaluated by calculating the inertia, which was defined as the ratio of the between-group variance to the global variance. The statistical significance of inertia for group separation was estimated using a Monte Carlo permutation test (1000 runs) and fixed to *P* < *0.05*. In each run, the simulated inertia was calculated. The distribution of simulated inertia values was then compared to the real inertia. A *P*-value was then calculated as the ratio of the number of simulations in which the simulated inertia was larger than the real inertia to the total number of runs.

## Additional Information

**How to cite this article:** Bacqué-Cazenave, J. *et al*. Social harassment induces anxiety-like behaviour in crayfish. *Sci. Rep.* **7**, 39935; doi: 10.1038/srep39935 (2017).

**Publisher's note:** Springer Nature remains neutral with regard to jurisdictional claims in published maps and institutional affiliations.

## Supplementary Material

Supplementary Movie 1

Supplementary Movie 2

Supplementary Movie 3

Supplementary Movie 4

Supplementary Figures and Legends

## Figures and Tables

**Figure 1:**
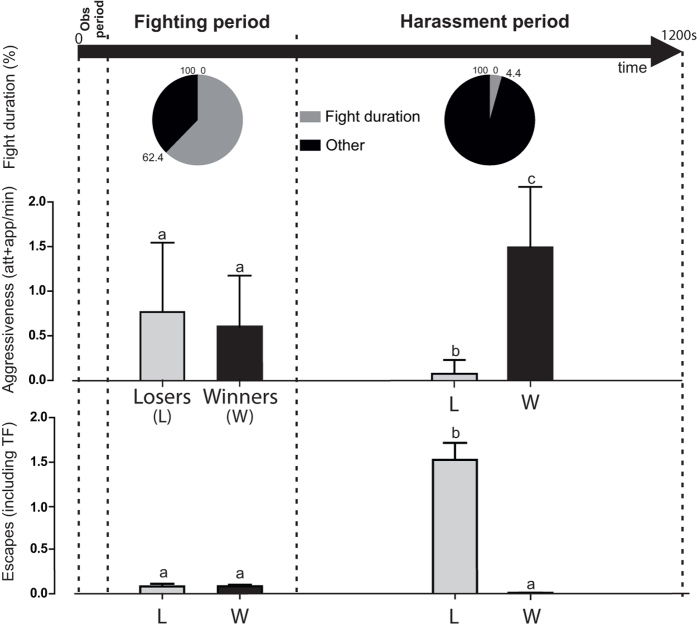
Social interactions of paired crayfish. Crayfish were paired in a fight arena (33 × 23 × 20 cm) for 20 minutes. Encounters started with an exploratory behaviour (*Obs period*, 56 ± 10 s, n = 23 pairs of crayfish). Then, a *fighting period* (372 ± 68 s) began with reciprocal aggressive behaviours (attacks and approaches, *att* + *app*) that turned into fights (fights duration was 232 ± 62 s i.e. 62.4% of *fighting period*) and ended with the retreat of one opponent (the loser, *L*). Then, a new period, called here *harassment period*, started (772 ± 64 s). During this period, the winner (*W*) aggressiveness increased significantly (from 0.59 ± 0.11 to 1.5 ± 0.14 att + app/min, two ways ANOVA, *P* < 0.0001, n = 23), while loser escaped with defensive behaviours (from 0.08 ± 0.03 to 1.5 ± 0.2 escapes, two ways ANOVA, *P* < 0.0001, n = 23). Consequently, fight duration dramatically decreased (34 ± 20 s, 4.4%, n = 23, *P* < 0.0001, Mann-Whitney test). Absence of identical letters above bars indicates a significant difference between results (Tukey’s multiple comparisons test).

**Figure 2:**
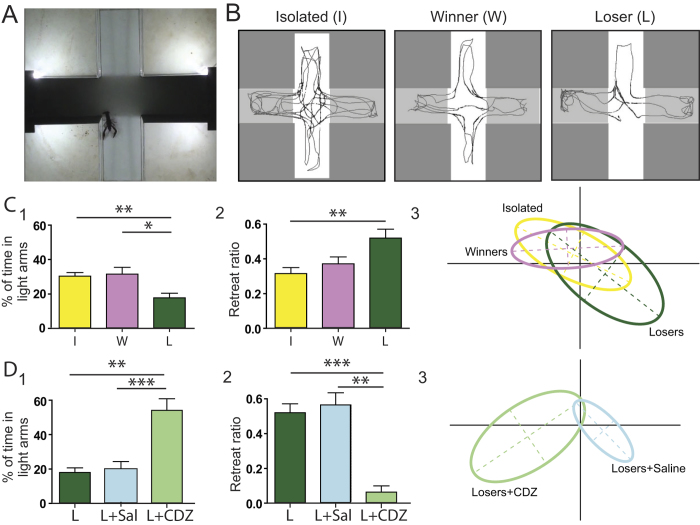
Social interactions elicited ALB. (**A**) Photographs of the dark/light plus maze. (**B**) Examples of typical crayfish routes tracked with the Ethovision software. Note that *isolated* (I) and *winner* (W) crayfish explored the entire maze while *loser* (L) was restrained to dark places. (**C**) The %of time in illuminated arms (C1,) decreased significantly (*P* < 0.01, Kruskal-Wallis followed by Dunn’s multiple comparison test) in losers (17.7 ± 2.7%, n = 27, yellow bar) as compared to winner (30.4 ± 2.1%, n = 27, *P*_LvsW_ < 0.05, pink bar) and isolated crayfish (31.6 ± 4%, n = 54, *P*_LvsI_ < 0.01, dark green bar). The retreat ratio (C2) significantly increased in losers (0.51 ± 0.05) as compared to isolated (0.31 ± 0.03, Kruskal-Wallis test followed by Dunn’s multiple comparison test, *P* < 0.01). Principal component analysis (PCA) showed that isolated and winners were two related groups (yellow and pink groups, *P* > 0.05, Monte-Carlo test). By contrast, losers (green group) were significantly separated from isolated and winners, (respectively *P* = 0.003 and *P* = 0.01, Monte-Carlo test). (**D**) Chlordiazepoxide (*CDZ*) abolished ALB in losers. % of time in illuminated arms (D1) was significantly higher in losers injected with CDZ after social interactions (*L* + *CDZ*, 53.8 ± 6.7%, n = 7, light green) as compared with losers injected to saline (*L* + *Sal*, 19.8 ± 3.9%, n = 15, light blue) and losers (*L*, 17.7 ± 2.7%, n = 27, dark green), respectively *P* < 0.001 and *P* < 0.01 (Dunn’s Multiple Comparison Test). Retreat ratio (D2) decreased in losers + CDZ (0.06 ± 0.03, n = 7) as compared with losers saline (0.56 ± 0.07, n = 15) and losers (0.52 ± 0.05, n = 27), respectively *P* < 0.01 and *P* < 0.001 (Dunn’s Multiple Comparison Test). Results in PCA analysis (D3) showed that *Losers* + *CDZ* and *Losers* + *Saline* are 2 separated groups (respectively light green and light blue groups, *P* = 0.001, Monte-Carlo test).

**Figure 3:**
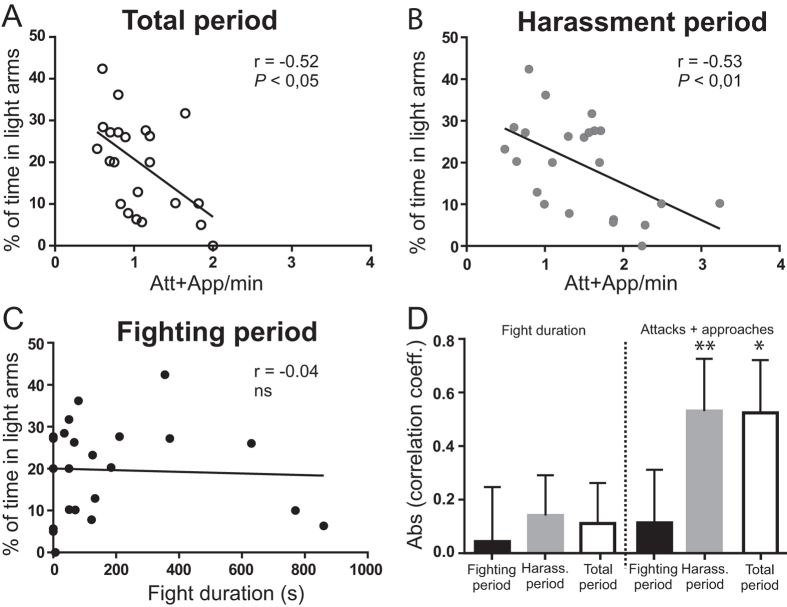
ALB is mainly correlated to harassment. (**A,B**) Correlations between the level of aggressiveness of the winner (attacks and approaches, *Att* + *App*) and the % of time in light arms of the loser during (**A**) the total period of pairing (white circle) or (**B**) the harassment period (grey circle). The correlations were significant (*r*, Pearson correlation coefficient, two-tailed P value, *P* < 0.01). (**C**) Absence of correlation between fight duration and the % of time in light arms during the *fighting period*. (**D**) Comparison of the absolute values *(Abs)* of correlation coefficients: on left side, between % of time in light arms and fight duration, 0.02 ± 0.20 (*fighting period*), 0.31 ± 0.19 (*harassment period*) and 0.15 ± 0.20 (*total period*). On right side, same analysis for the number of attacks and approaches per min (n = 23) during total period of matches (*Total period*, 0.59 ± 0.14, white bar, *P* < 0.01); harassment period (*Harass. period*, 0.55 ± 0.20, gray bar, *P* < 0.01) and fighting period (*Fighting period*, 0.06 ± 0.15, dark bar, p > 0.05). The two significant correlations observed above (right side, *total period* and *harassment period*) were not significantly different between them (*P* = 0.95, Steiger’s Z-test.) indicating that harassment is responsible for the main part of social ALB.

**Figure 4:**
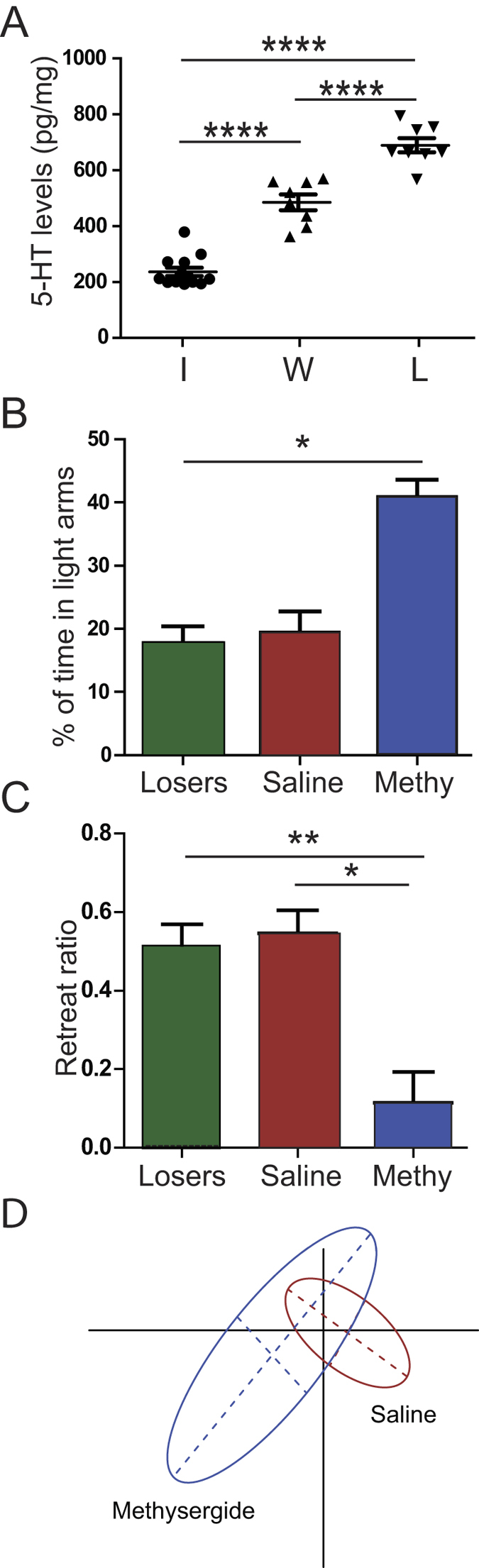
5-HT is involved in social ALB. (**A**) HPLC measurements showed that 5-HT levels in crayfish brain are higher after social interaction (isolated crayfish (*I*, n = 13) 236 ± 15 pg/mg, Winners (*W*, n = 8) 486 ± 28 pg/mg and losers (*L*, n = 8) 691 ± 25 pg/mg, ANOVA, *P* < 0.001). Moreover, 5HT levels in losers are systematically higher than those of the corresponding winners (Turkey’s multiple comparison test, p < 0,0001). (**B**) %of time in light arms increased if methysergide (*Methy*, n = 6) is injected before encounter (35 ± 4%) as compared with losers (Losers, n = 27, 17.7 ± 2.7%, Dunn’s multiple comparison test, *P* < 0.05) and crayfish injected with saline before encounter (Saline, n = 8, 19.4 ± 3%). (**C**) Similarly, retreat ratio decreased in methysergide group (0.16 ± 0.09, n = 6) in comparison with losers (0.52 ± 0.05, Dunn’s multiple comparison test, *P* < 0.01) or saline (0.55 ± 0.06, Dunn’s multiple comparison test, *P* < 0.05). (**D**) A global study with PCA analysis confirmed that injection of methysergide, a wide-spectrum 5-HT antagonist, prevented the onset of ALB. Indeed, Saline losers (Red group) and Methysergide losers (blue group) groups were significantly different (*P* < 0.009, Monte-Carlo test).
