# Supplementary material for: Social harassment induces anxiety-like behaviour in crayfish
Source: Sci Rep. 2017 Jan 3;7:39935. doi: 10.1038/srep39935 (PMC5206779; doi:10.1038/srep39935)
Supplement: Supplementary Figures and Legends [file srep39935-s5.doc]

**Supplementary Materials**

**Social harassment induces anxiety-like behaviour in crayfish**

by

**Julien Bacqué-Cazenave, Daniel Cattaert,**

**Jean-Paul Delbecque* and Pascal Fossat***

INCIA - Institut de Neurosciences Cognitives et Intégratives d'Aquitaine, University of Bordeaux, CNRS UMR 5287,146 Rue Leo Saignat, 33076 Bordeaux, France.

*The two last authors share seniority.

Corresponding author: Pascal Fossat, PhD, HDR.

Present address: IINS, Institute for Interdisciplinary Neurosciences, University of Bordeaux, CNRS UMR 5297, 146 Rue Léo Saignat, 33076 Bordeaux, France.

Email: [pascal.fossat@u-bordeaux.fr](mailto:pascal.fossat@u-bordeaux.fr)

Sup figure legends:

Sup Fig 1: **Principal component analysis (PCA) of the seven behavioral variables measured in experimental groups**. A: Contribution of the uncorrelated variables to the variances of the first and second components. B: Projection of group barycenters into the plane of the first and second components.

Sup Fig 2: **Statistical comparison of groups used in the PCA.** A) Examples of Monte-Carlo analysis of several groups. The distribution of simulated ratio values or simulated inertia (grey histograms) obtained with 1000 runs is compared to the experimental ratio value (*exp ratio*, vertical line labeled with filled diamond). In examples A1, A2 and A3, the experimental inertia is outside the simulated distribution (*P*<0.01), whereas in example A4, experimental ratio is not different from random (*ns*). B) Results of Monte-Carlo test for all groups. Grey values represent groups not significantly different (*P*>0.05).

Sup Fig 3: **ALB is mainly correlated to harassment.** Same legend as Fig.3 for retreat ratios

Sup Table S1: **Variables measured in the aquatic dark/light plus maze and analyzed in PCA**

| Name of variables | Definition | Measurement | Range |
| --- | --- | --- | --- |
| Distance walked | Distance walked in the entire arena. | cm | ≥0 |
| % light | (Time spent in light/ total time)*100 | % | 0-100 |
| Latency to first light | Time from start time to first entry in a light arm | Second (s) | 0-600 |
| Latency to first dark | Time from start time to first entry in a dark arm | Second (s) | 0-600 |
| Mean time per visit light | Time in light arms/mean number of entries | Second (s) | ≥0 |
| Mean time per visit dark | Time in dark arms/mean number of entries | Second (s) | ≥0 |
| Retreat Ratio  (RR) | Number of retreats/total number of attempts | NA | 0-1 |

Table S1 NA : non applicable

Sup Video 1: example of observation period (extract).

Sup Video 2: example of fighting period (extract).

Sup Video 3: example of end of fighting period by loser’s decision to retreat.

Sup Video 4: example of harassment period (extract).

Supplementary Figures :
